# A statistical model-building perspective to identification of MS/MS spectra with PeptideProphet

**DOI:** 10.1186/1471-2105-13-S16-S1

**Published:** 2012-11-05

**Authors:** Kelvin Ma, Olga Vitek, Alexey I Nesvizhskii

**Affiliations:** 1 Department of Statistics, Purdue University, 250 N. University Street, West Lafayette, Indiana, USA; 2 Department of Computer Science, Purdue University, 305 N. University Street, West Lafayette, Indiana, USA; 3 Department of Pathology, University of Michigan, 4237 Medical Science I, Ann Arbor, Michigan, USA

## Abstract

PeptideProphet is a post-processing algorithm designed to evaluate the confidence in identifications of MS/MS spectra returned by a database search. In this manuscript we describe the "what and how" of PeptideProphet in a manner aimed at statisticians and life scientists who would like to gain a more in-depth understanding of the underlying statistical modeling. The theory and rationale behind the mixture-modeling approach taken by PeptideProphet is discussed from a statistical model-building perspective followed by a description of how a model can be used to express confidence in the identification of individual peptides or sets of peptides. We also demonstrate how to evaluate the quality of model fit and select an appropriate model from several available alternatives. We illustrate the use of PeptideProphet in association with the Trans-Proteomic Pipeline, a free suite of software used for protein identification.

## Introduction

In mass-spectrometry shotgun proteomics, the first phase of analysis is the identification of peptides in complex biological mixtures digested by enzymes such as trypsin. Dependent on the peptides in the biological mixture, an experiment will produce a certain number of spectra (call it *N*). MS/MS spectra are individually matched to peptides by searching through a database of peptides predicted from the genome of the organism. The way the searches are performed can be constrained using different search parameters, such as the number of tryptic termini (NTT), number of missed cleavages (NMC) or the mass difference of the observed precursor ion mass and the weight of the theoretical peptide (Δ*M*).

We will discuss PeptideProphet in the context of two database search algorithms: SEQUEST [1] and Tandem with the k-score plugin [2,3]. SEQUEST attempts to determine a direct correlation between an observed spectrum and sequences of amino acids in a protein sequence database. Typical quantities associated with SEQUEST include: *XCorr*, Δ*Cn*, *SpRank*. Typical quantities associated with Tandem with the k-score plugin include: *logDot *(logarithm of dot product between observed and theoretical spectrum) and Δ*Dot*. PeptideProphet can be used with any database search algorithm that returns a quantitative score.

Given a database search algorithm, every spectrum that is observed will be scored against the peptides in the database. For each spectrum, the highest scoring peptide (depending on the scoring criterion) is typically chosen as the best match. The best match is the potential peptide sequence that generated its corresponding observed spectrum. Thus, we have *N *spectra that have been matched to a peptide and we will refer to these spectra as identified spectra.

The necessity of PeptideProphet arises because the spectra are subject to noise making it difficult to determine if the peptide that it is matched to is correct. The spectrum itself is generated from a peptide sequence and peaks can be missing or reduced in intensity. Because the spectrum that is being generated is subject to noise the database-based criterion will vary when comparing theoretical spectra to observed spectra. Additionally, when searching the database, the correct peptide sequence may be absent. Because of this noise, how do we determine confidence in an identified spectrum? Traditional standards (such as just accepting all above *XCorr *> 2.5) does not reflect the quality of the identification. Such a rule may accept too many incorrectly identified spectra. Thus, statistical inference is needed to model the presence of noise.

PeptideProphet [4] is a post-processing and rescoring algorithm for determining confidence in identified spectra found using a database search. PeptideProphet is one of the first methods for the assessment of confidence. It is based on a probability model and an Empirical Bayesian approach to model fitting. It is now not a single model, but a family of models [5].

The overview of PeptideProphet is as follows:

1. Rescoring: produce a score which reflects the quality of an identified spectrum, while summarizing multiple quantities, such as *XCorr *and Δ*Cn *or *logDot *and Δ*Dot*. The rescoring separates incorrectly and correctly identified spectra scores as much as possible.

2. Modeling: produce a probability-based model for the distribution of correctly and incorrectly identified spectra. The model must be then fit to the scores of all identified spectra.

3. Evaluation of the Quality of Fit: determine how well the scores fit the probability-based model.

4. Inference

(a) Evaluation of confidence in individual identified spectra using the posterior probability.

(b) Evaluation of confidence in sets of identified spectra: produce a cutoff on the scores to determine a set of correctly identified spectra while controlling the False-Discovery Rate, defined as the expected proportion of false positives.

We will first discuss the basic version of PeptideProphet and then discuss the three extensions.

## Materials

### Human plasma dataset

This dataset uses the first LC-MS/MS replicate file from the Western Consortium of the National Cancer Institute's Mouse Models of Human Cancer [6]. The data was obtained using the Multiple Affinity Removal System and was matched using a semitryptic SEQUEST search against an IPI human protein database allowing a 3 Dalton mass tolerance and 0-1 missed cleavage sites. More details on the spectra can be seen in [7].

### Controlled mixture

This dataset uses spectra generated from a linear ion trap Fourier transform instrument that was published in [8]. In particular the spectra from Mixture 3 was used, where 16 known trypsin-digested proteins from different mammals were analyzed. Spectra were also matched using a semitryptic SEQUEST search against a database file with the 16 known proteins concatenated with human influenza proteins allowing a 3 Dalton mass tolerance and 0-2 missed cleavage sites. Matches to human influenza proteins are known to be incorrect. More details on the dataset can be seen in [8].

## Methods

### Statement of the problem from a statistical perspective, and terminology

Every statistical approach requires the definition of the following components in the problem:

1. PeptideProphet works with the observed spectra as the *experimental unit *where we have *N *observed spectra with *N *being generally large (in the thousands or more). Since the number of spectra *N *is typically very large, the identified spectra can be viewed as the underlying *population*.

2. An observed score is interpreted as a test statistic. In statistics the summarized score *S *is called a *test statistic *because it is the function of the observed experimental unit that is being used to answer our hypotheses.

3. PeptideProphet assumes that the test statistic comes from a mixture of two distributions: one from the distribution of correct identifications, and the other from the distribution of the incorrect identifications. The distributions may be characterized by a few parameters (parametric) or many parameters (semi or non-parametric).

4. The goal of PeptideProphet is to test two competing *hypotheses *for each identified spectrum. Let *T_i _*be the true status of identified spectrum *i *where *T_i _*= 0 indicates that the identified spectrum was incorrectly identified and where *T_i _*= 1 indicates that the identified spectrum was correctly identified. We then wish to compare:

$$H_{0i}:T_{i}=0\;\;(\text{null}\;\text{hypothesis)}\;\text{versus }H_{\text{1}i}:T_{i}=1\;\;\left(\text{alterative}\;\;\text{hypothesis}\right)$$

5. Inference: confidence is determined for individual spectra or sets of spectra.

• If the researcher is interested in a set of spectrum identifications, the False Discovery Rate should be controlled.

We determine the confidence in a set of spectra by controlling the False Discovery Rate. The False Discovery Rate, given a cutoff *δ*, is the expected proportion of all scores *S_i _*>*δ *that are truly incorrect (the proportion of accepted identified spectra that are false positives). This situation is synonymous to performing *N *multiple hypothesis tests where $FDR=E\;\left[\frac{V}{R}|R>0\right]\;P\left(R>0\right)$ using the values in Table 1. *P *(*R *> 0) is assumed to be 1 when we perform many tests (*N *is large). The False Discovery Rate is the expected proportion of incorrectly rejected null hypotheses out of the total rejected hypotheses. For a given cutoff if we were to repeat the experiment an infinite number of times and use the same cutoff each time the expected False Discovery Rate is the average proportion of incorrectly identified and accepted spectra out of the total number of incorrectly identified spectra.

**Table 1:** Table of multiple hypothesis testing quantities

	# Not Rejected	# Rejected	Total
# True Nulls	*U*	*V*	*N*_0_
# True Alternatives	*T*	*S*	*N - N*_0_

Total	*N - R*	*R*	*N*

Table 1: *U*, *V*, *T*, and *S *correspond to the number of true negatives, false positives, false negatives, and true positives respectively.

An alternative confidence rate that is rarely used is the False Positive Rate (FPR). The False Positive Rate, given a cutoff *δ *is the expected proportion of all truly incorrectly identified spectra that are considered to be correctly identified. From the terms in Table 1 it is represented by $FPR=E\;\left[\frac{V}{N_{0}}|N_{0}>0\right]\;P\;\left(N_{0}>0\right)$

Many users prefer the q-value which is the minimum False Discovery Rate required for a score *s_i _*to be considered significant. It is represented by $qvalue\left(s_{i}\right)=inf_{\left\{\Gamma :s_{i}\in \Gamma \right\}}FDR\left(\Gamma \right)$, where Γ represents the set of all possible cutoff scores [9]. This confidence measure is used to describe a score *s_i _*at a single point but examines the False Discovery Rate of all possible scores. Unlike the False Discovery Rate, the q-value is a monotonic quantity with respect to the score cutoff.

• If the researcher is interested in specific spectrum identifications the posterior error probability is most commonly used as it quantifies the confidence of a single identified spectrum.

The posterior error probability represents $P\left(T_{i}=0|S_{i}\right)$ which we also denote as *PEP*. In other words using a probability model for *S_i_*, we can find the probability of an identified spectrum being incorrect given its test statistic. Note that we can also calculate $P\left(T_{i}=1|S_{i}\right)=1-P\left(T_{i}=0|S_{i}\right)$ which is the probability of an identified spectrum being correct given its test statistic. The posterior error probability is also called the local false discovery rate (locfdr) [10,11].

Alternatively the p-value can be used. If *s_i _*is the *i*th observed score then the p-value represents $P\left(S_{i}\geq s_{i}|{H_{0}}_{i}\right)$, or the probability of observing a score equal to or greater than *s_i _*assuming that the *i*th identified spectrum was incorrectly identified. The p-value is similar to the FPR in that the p-value is the probability of observing a score equal to or greater than *s_i _*assuming that it is one of the *N*_0 _truly null hypotheses.

### For each spectrum, PeptideProphet establishes a score reflecting the quality of an identified spectrum

First each spectrum (experimental unit) is observed and potentially identified using a database-based criterion (*XCorr*, Δ*Cn*, *logDot*, Δ*dot*, etc.), PeptideProphet rescores the identified peptide with a discriminant function, using the database-based criterion as the covariates for fitting the discriminant function. The goal is to fit a function that separates correct scores from incorrect scores. If *S_i _*is the summarized score for the *i*th identified spectrum from a SEQUEST search result, a discriminant function produces a linear function *f*:

(1) $$S=f_{SEQUEST}\left(XCorr,\Delta C_{n},SpRank\right)=\beta_{0}+\beta_{1}XCorr+\beta_{2}\Delta C_{n}+\beta_{3}SpRank$$

such that *S *> 0 for correctly identified spectra and *S *< 0 for incorrectly identified spectra.

If *S_i _*is the summarized score for the *i*th identified spectrum from a Tandem search result, a linear discriminant function is used but with different coefficients:

(2) $$S=f_{TANDEM}\left(XCorr,\Delta C_{n},SpRank\right)=\beta_{0}+\beta_{1}logDot+\beta_{2}\Delta Dot$$

In the basic version of PeptideProphet the *β*'s are estimated empirically from a controlled mixture and are dependent on the precursor ion charge (i.e. a separate discriminant function was trained for 1+, 2+, 3+ precursor ion charges).

### PeptideProphet relates observable and unobservable quantities via a joint probability distribution

PeptideProphet relates scores *S_i _*to parameters via a *sampling distribution *of the test statistic under *H*_0*i *_and *H_ai_*. All scores *S_i_*'s are independent and identically distributed (iid). The sampling distribution of *S_i _*is assumed to follow a *Normal*(*μ*, *σ*) distribution if the identified spectrum is correct (*T_i _*= 1) and *Gamma*(*α*, *β*, *γ*) distribution if the identified spectrum is incorrect (*T *= 0). Notationally we have that $p\left(S_{i}|T_{i}=0\right)\sim Gamma\left(\alpha ,\beta ,\delta \right)$ and that $p\left(S_{i}|T_{i}=1\right)\sim Normal\left(\mu ,\sigma \right)$. Note that other forms of the distribution of scores for incorrect identifications such as the Gumbel distribution are often used with no effect on the theory presented here. Among all identified spectra an additional parameter *π*_0 _is used to represent the overall proportion of incorrect identifications of identified spectra in the population. This formulation results in a 2-group mixture model similar to what is established by Efron [10] where we may write that

(3) $$S_{i}\sim P\left(T_{i}=0\right)p\left(S_{i}|T_{i}=0\right)+P\left(T_{i}=1\right)p\left(S_{i}|T_{i}=1\right)=\pi_{0}f_{T=0}+\left(1-\pi_{0}\right)f_{T=1}$$

The last equality is due to the fact that all scores are independent and identically distributed (iid). Due to different discriminant functions being used for each charge, a different sampling distribution and set of parameters are produced for each precursor ion charge (we will refer to this simply as the charge).

There may be additional information available, such as the NTT (number of tryptic termini), NMC (number of missed cleavages), and Δ*M *(delta mass) that can be used to improve the estimation of the sampling distribution of the identified spectra [7,12,13]. For example, the use of NTT = 0 in unconstrained searches often leads to improved estimation of the parameters even in lower quality datasets [5]. This is incorporated into the model above by assuming the existence of additional distributions for incorrect and correct identifications:

(4) $$\left(S_{i},NTT_{i},NMC_{i},\delta M_{i}\right)\sim \pi_{0}f_{T=0}f_{T=0}{,}_{NTT}f_{T=0,NMC}f_{T=0}{,}_{\Delta M}+\pi_{1}f_{T=1}f_{T=1}{,}_{NTT}f_{T=1}{,}_{NMC}f_{T=1}{,}_{\Delta M}$$

Note that the density functions of *f_T_*_=0_, *_NTT_*, *f_T_*_=0, *NMC*_, *f_T_*_=0_, _Δ*M*_, *f_T_*_=1, *NTT*_, *f_T_*_=1_, _*NMC*_, and *f_T_*_=1_, _Δ*M *_are discrete. It is assumed, conditional on the identified spectrum being incorrect or correct, that the members of (*S_i_*, *NTT_i_*, *NMC_i_*, *δM_i_*) are independent, as shown above.

### PeptideProphet estimates parameters of interest in an Empirical Bayesian approach

PeptideProphet is considered an Empirical Bayesian approach because it uses each identified spectrum twice: once to estimate via the Expectation-Maximimzation [14] algorithm the parameters of the sampling distribution (π_0_, *μ*, *σ*, *α*, *β*, and *γ*) and second to estimate the confidence in the correctness of an identified spectrum. The EM-algorithm iterates between two steps, called the E-step and the M-step in order to estimate the value of model parameters. With a large enough set of identified spectra (say 100), the EM-algorithm will always converge [14]. The algorithm starts with initial values of model parameters π_0_, *μ*, *σ*, *α*, *β*, and *γ*.

In the E-step, given the estimated values of the model parameters, the probability of each score being correct (or incorrect) is calculated. Given a single observed score *s_i _*and its correctness status *T_i_*, usage of Bayes Theorem yields $P\left(T_{i}=0|S_{i}=s_{i}\right)=\frac{P\left(T_{i}=0\right)p\left(S_{i}=s_{i}|T_{i}=0\right)}{P\left(T_{i}=0\right)p\left(S_{i}=s_{i}|T_{i}=0\right)+P\left(T_{i}=1\right)p\left(S_{i}=s_{i}|T_{i}=1\right)}$ which corresponds to the ratio of the Gamma density scaled by *π*_0 _over the sum of the Gamma and Normal densities scaled by *π*_0 _and 1 - *π*_0 _at score *s_i_*.

In the M-step, given estimated membership probabilities $P\left(T_{i}=0|S_{i}=s_{i}\right)=p_{i}$ for each score *s_i_*, the model parameters are re-estimated by finding the values with the maximum likelihood. The estimate of *π*_0 _is $\frac{\sum_{i=1}^{N}p_{i}}{N}$. For the Normal distribution the estimates of *μ *and *σ*^2 ^are:

$$\hat{\mu }=\frac{\sum_{i=1}^{N}\left(1-p_{i}\right)s_{i}}{\sum_{i=1}^{N}\left(1-p_{i}\right)}$$

$${\hat{\sigma }}^{2}=\frac{\sum_{i=1}^{N}\left(1-p_{i}\right){\left(s_{i}-\hat{\mu }\right)}^{2}}{\sum_{i=1}^{N}\left(1-p_{i}\right)}$$

For the Gamma distribution, the estimate of *γ *is simply the minimum of the scores *s_i_*, *i *= 1, ..., *N*. In order to estimate *α *and *β *let $m_{1}=\frac{\sum_{i=1}^{N}p_{i}\left(s_{i}-\hat{\gamma }\right)}{\sum_{i=1}^{N}p_{i}}$ and $m_{2}=\frac{\sum_{i=1}^{N}p_{i}{\left(s_{i}-\hat{\gamma }-m_{1}\right)}^{2}}{\sum_{i=1}^{N}p_{i}}$. Then the estimates of *α *and *β *are

$$\hat{\alpha }=\frac{m_{1}^{2}}{m_{2}}$$

$$\hat{\beta }=\frac{m_{1}}{m_{2}}$$

**Figure 1:**
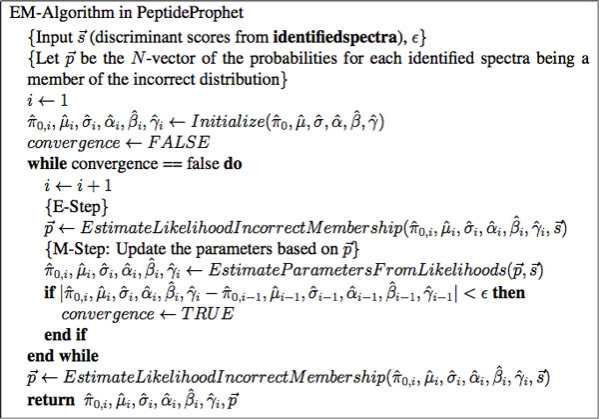
**Pseudocode of the EM-algorithm for iteratively estimating model parameters and membership probabilities**.

**Figure 2:**
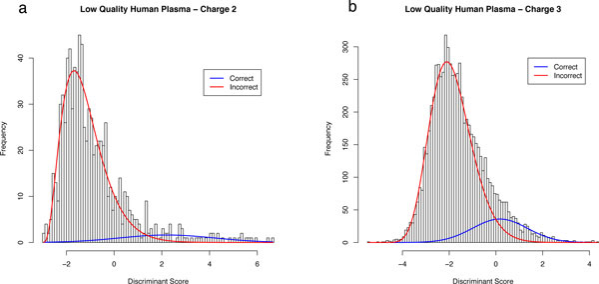
**PeptideProphet fits on the Human Plasma Dataset**. PeptideProphet fits on the Human Plasma Dataset with Tandem Scores on charges 2 (left) and 3 (right). The blue and red curves correspond to the fitted frequency curves of the correct (Normal) and incorrect (Gamma) distributions. The Charge 2 fits yields a mixture distribution with a much stronger separation than the fit to Charge 3.

Due to the speed of the algorithm in working with only two mixture components, the process of the E and M-step can be iterated repeatedly until the model parameters do not change by a specified *ε *where *ε *is a small number, such as 0.0001. The algorithm then outputs estimated parameters of *α*, *β*, *δ*, *μ *and *σ*, as well as the estimate of *π*_0 _(denoted with hats when estimates). The algorithm is detailed in Figure 1. Figures 2b and 2a shows two fits of PeptideProphet to the Human Plasma dataset of charges 2 and 3. Note that the EM algorithm can be substituted for alternative algorithms such as the Method of Moments.

### Evaluation of the quality of fit of PeptideProphet

Deviations of the assumptions, or a low number of identified spectra can lead to an inadequate or unstable model fit and incorrect conclusions. This can be diagnosed by visual inspection, and also by the bootstrap. We recommend using visual inspection over goodness of fit tests as tests do not explore the specific fitting issues that may influence subsequent inference of the identified spectra. In fact goodness of fit tests simply attempt to summarize the goodness of fit into one summary statistic whereas we are typically interested in the fit at certain locations of the mixture distribution. There are several visual attributes of the mixture distribution that researchers should be aware of and some remedies for them.

*Do the empirical scores follow the fitted curves well? *Particular attention needs to be paid to the tails of the distributions, especially the right tail of the distribution of scores of incorrect identifications (red) and the left tail of the distribution of scores of correct identifications (blue). This is often of most interest to researchers as the identified spectra in these regions are considered to be borderline correct or incorrect. In the case of Figure 2b the curves fit the histogram well but in Figure 2a there are many mismatches in the bars and the fitted curves. The culprit of these mismatches is likely due to the small number of spectra. The right portion of the Normal distribution is fit with approximately only 30 spectra. If the data is comprised of a large number of spectra but is deviating from the fitted curves, robust procedures can also be considered and will be discussed later.

*Do the curves highly overlap? *Although high overlap does not necessarily indicate a poor fit it will lead to smaller sets of confidently identified spectra. Overlaps that occur in situations of highly constrained searches can be remedied with techniques in later sections. Overlap in the case of a small number of spectra (Figure 2a) may be remedied by artificially adding observations using decoys which will also be subsequently demonstrated.

An issue that is not commonly addressed however is the number of identified spectra available to fit the mixture model. The number of identified spectra required to fit a reliable model depends highly on the separation and the form of the observed scores. A statistical approach to examine the stability of the fitted model can be done via the bootstrap.

Bootstrapping can be performed by sampling with replacement *B *samples (spectra) where each is of size *N *from the original dataset. At least 100 to 500 bootstrapped samples are recommended. For each bootstrapped sample *b*, we can refit the PeptideProphet model to receive bootstrapped estimates of ${\hat{\pi }}_{0,b}^{*}$, ${\hat{\mu }}_{b}^{*}$, ${\hat{\sigma }}_{b}^{*}$, ${\hat{\alpha }}_{b}^{*}$, ${\hat{\beta }}_{b}^{*}$, and ${\hat{\gamma }}_{b}^{*}$. The bias, variance, and mean squared error (MSE) of the procedure used to estimate a parameter can be found using the bootstrapped estimates. In the case of *μ*, the bootstrap bias estimate is $\hat{bias}=\frac{\sum_{b=1}^{B}{\hat{\mu }}_{b}^{*}}{B}-\hat{\mu }$. Large biases imply that the estimation procedure is systematically over or underestimating the true value of a parameter. Note that as *B *increases the bias does not move towards 0. The bootstrap variance estimate is defined as $\hat{variance}=\frac{\sum_{b=1}^{B}\left({\hat{\mu }}_{b}^{*}-\frac{\sum_{b=1}^{B}{\hat{\mu }}_{b}^{*}}{B-1}\right)}{B}$. Smaller variability is desired. The bias and variability of an estimation procedure is often summarized using the mean squared error, which is $\hat{MSE}=\hat{variance}+{\hat{bias}}^{2}$.

**Figure 3:**
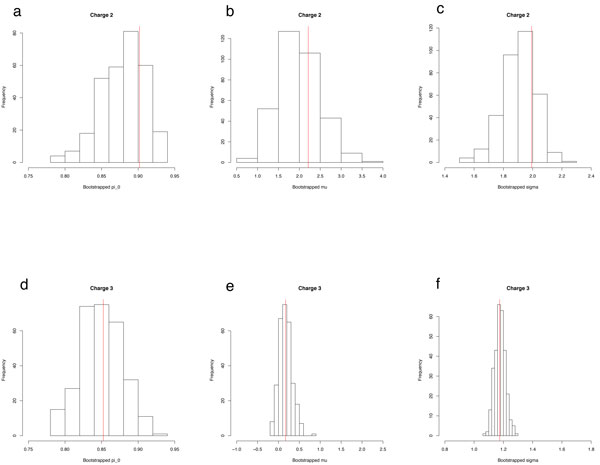
**Bootstrapped samples of **π_0_, *μ ***, and ***σ ***for Charges 2 and 3 of the Human Plasma data**. The original estimates are marked by the vertical line. The length of the horizontal axes are equal for the plots of a particular parameter. The Charge 2 distributions are slightly skewed compared to Charge 3 distributions and the mean squared errors are much greater in Charge 2 distributions. The variability of the Charge 2 distributions are visibly much greater indicating unstable estimates.

Three hundred bootstrapped samples for the Human Plasma data for charges 2 and 3 were performed and the bootstrapped estimates for *π*_0_, *μ*, and *σ *are shown in Figure 3. Although the means of the bootstrapped distribution are close to the original estimates (marked in red) the bootstrapped distributions for these parameters are more skewed for Charge 2 than for Charge 3. Additionally the variance of the bootstrapped estimates is significantly greater in the Charge 2 case for *μ *and *σ *showing how unstable the estimates for the Charge 2 distribution given the small number of identified spectra.

**Figure 4:**
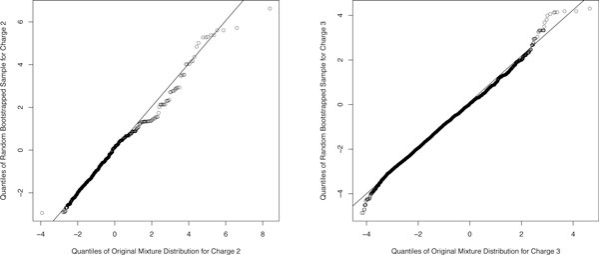
**Quantile-to-quantile plot comparing the quantiles of the original mixture distribution of the Human Plasma data**. Quantile to quantile plot comparing the quantiles of the original mixture distribution of the Human Plasma data for Charges 2 (left) and 3 (right) compared to the quantiles of randomly bootstrapped samples. The quantile-to-quantile plot for Charge 2 shows more deviation in quantiles due to the low number of identified spectra in the score range between 2 and 8.

The mean squared error summarizes the overall deviation of parameter estimates from *B *bootstrapped samples to the original estimates. The experimenter may also view the deviations that occur between the original sample and a single bootstrapped sample. Although a histogram of both samples would suffice, a quantile-to-quantile plot is an easy-to-read plot that exemplifies the deviations between the two plots. The quantile-to-quantile plot plots the quantiles of one distribution versus the matched quantiles of the other. For example if there are 10 values in two datasets the quantile-to-quantile plot would display the 10, 20, 30,..., and 100th percentiles of one distribution matched with the respective 10, 20, 30, ..., and 100th percentiles of the second distribution. Distributions that are alike should result in a quantile-to-quantile plot that is linear. Deviations from linearity at different quantiles in the plot imply differences between the two distributions at those associated quantiles. Although no quantile-to-quantile plot will be perfectly linear the plot should not deviate much at the center and right portions of the plot as the accuracy of the estimated confidence of identified spectra relies heavily upon a good fit at these locations. The quantile-to-quantile plot for Charge 2 in Figure 4 displays the deviation in quantiles of the original mixture distribution and the quantiles of a random bootstrapped sample. The deviations noticeably occur in the right half of the plot which corresponds to the right portion of the axis in Figure 2a indicating that the instability of the estimate is due to the right half of the plot. More specifically, it is due to the low number of identified spectra in this area of the plot.

### Estimating the confidence of spectrum identifications

#### Estimating the confidence of a set of spectrum identifications

In order to determine the correctness of the spectrum identifications, a decision rule is defined where any spectrum identification with a score above *δ *is concluded to be correct. In many experiments we are interested in the statistical properties of the list of spectrum identifications with scores above *δ*.

**Figure 5:**
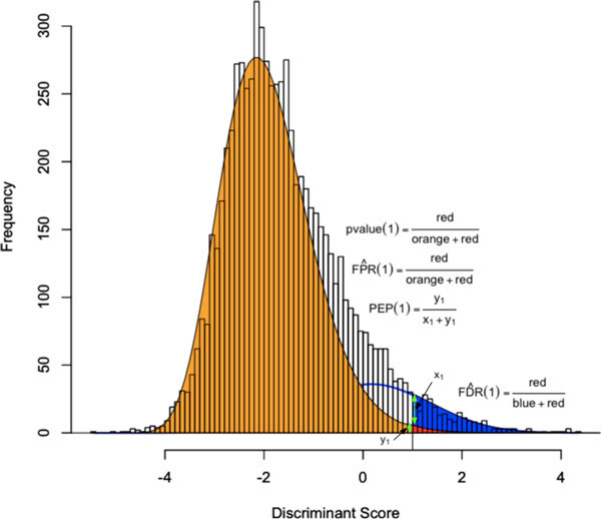
**The PeptideProphet fit to the Human Plasma dataset of Tandem scores of Charge 2**. The PeptideProphet fit to the Human Plasma dataset of Tandem scores of Charge 2 with fitted frequency curves from Figure 2b. The four confidence measures of the Posterior Error Probability (PEP), p-value, False Discovery Rate (FDR), and False Positive Rate (FPR) are shown at a score of 1. The Posterior Error Probability at 1 is 0.156 and the estimated False Discovery Rate is 0.083. The p-value and FPR are equivalent and equal to 0.004. In the formula for the estimated FDR, *red *is the estimate for *V *from Table 1 while *blue *combined with *red *is an estimate for *R *from Table 1.

In order to estimate the False Discovery Rate given a decision rule cutoff two approaches may be used. Because all scores are assumed to follow the same fitted distribution the False Discovery Rate can be estimated with $F\hat{D}R\left(t\right)=\frac{{\hat{\pi }}_{0}P\left(S>t|T=0\right)}{{\hat{\pi }}_{0}P\left(S>t|T=0\right)+\left(1-{\hat{\pi }}_{0}\right)P\left(S>t|T=1\right)}$[15]. This can be seen by using the areas under the colored curves in Figure 5. In a second approach, PeptideProphet traditionally estimates the False Discovery Rate by interpreting the posterior error probabilities as local false discovery rates [10,11]. The estimated overall False Discovery Rate at point *t *is the average of the estimated local false discovery rates of identified spectra with scores greater than $t:F\hat{D}R\left(t\right)=\frac{\sum_{s_{i}\geq t}PEP_{i}}{\left\{\#s_{i}:s_{i}\geq t\right\}}$.

The False Positive Rate for a cutoff *t *can also be estimated using the area under the fitted frequency curve of the distribution of scores for incorrect identifications as seen in Figure 5. Mathematically this is equivalent to the p-value, or $F\hat{P}R\left(t\right)=P\left(S>t|H_{0}\right)$ since each incorrect score follows the same distribution. Note that the False Positive Rate ignores the distribution of scores for correct identifications.

The estimation of the q-value at a specific point *ρ *requires the estimation of the False Discovery Rate at every point *s_i _*from *i *= 1, 2, ..., *N*. The q-value for a point *ρ *is the minimum False Discovery Rate among all points *s_i _*such that *s_i _*≤ *ρ*. The estimated False Discovery Rate can be found using the model-based estimates or by interpreting each posterior error probability as a local false discovery rate. The q-value is often useful if a monotonically increasing error rate is desired for decreasing cutoff values. For example, in the case of Figure 5 suppose the experimenter was only interested in scores around 4. Using model-based estimates, the estimated False Discovery Rate with a cutoff at 4 is 0.01503874 but the estimate of the False Discovery Rate with a cutoff at 3.8 is 0.01489971 suggesting that the error rate is lower for a lower cutoff value. To avoid this issue, the q-value can be used as it finds the minimum False Discovery Rate at each cutoff value. The q-value at 4 is 0.01489939 (found using increments of 0.01 searching all FDR values from -4 to 4).

#### Estimating the confidence of an individual spectrum identification

We now discuss the estimation of the posterior error probability and the p-value. These measures are properties of a single spectrum and are synonymous to performing a single hypothesis test. In Figure 5 the posterior error probability and p-value only apply to spectra at a single point.

According to Bayes Theorem the posterior probability of *T_i _*= 0 (our hypotheses of interest) given its test statistic is $P\left(T_{i}=0|S_{i}=s_{i}\right)=\frac{P\left(T_{i}=0\right)p\left(S_{i}=s_{i}|T_{i}=0\right)}{p\left(S_{i}=s_{i}\right)}$. Following the Empirical Bayesian step where parameters are estimated we have that $P\left(T_{i}=0|S_{i}\right)=\frac{{\hat{\pi }}_{0}f_{T=0}\left(s_{i}\right)}{{\hat{\pi }}_{0}f_{T=0}\left(s_{i}\right)+\left(1-{\hat{\pi }}_{0}\right)f_{T=1}\left(s_{i}\right)}$. Because the posterior error probability is equivalent to the local false discovery rate we also have that $locfdr=P\left(T_{i}=0|S_{i}\right)$.

The p-value is estimated as $P\left(S_{i}>s_{i}|H_{0i}\right)$ which is the right tail-end of the Gamma density past *s_i_*.

The posterior error probability may be preferred over the p-value because it also yields an estimate for the probability of an identified spectrum to being correct (1 - *PEP*). The advantage of the p-value is that it only requires the use of the distribution of scores for incorrect identifications as it ignores the distribution of scores for correct identifications. Notice that in Figure 5 the p-value at a score of 1 is a low value of 0.004 but that the Posterior Error Probability at 1 is a much higher value at 0.156.

### PeptideProphet can use a decoy database to estimate the parameters of the distributions of scores for incorrect identifications

When there is significant overlap between the two density functions or a low number of identified spectra it is difficult for the EM-algorithm to estimate *π*_0 _and the parameters of the Gamma and Normal distributions. In this case PeptideProphet employs the Target-Decoy approach to better estimate the Gamma distribution. We first describe the two forms of Target-Decoy: the concatenated strategy and the separate strategy [16,17]. The objective of both strategies is to introduce decoys in order to estimate the error rate since decoys are known to be incorrectly identified spectra. Reversed sequences (decoy sequences) are commonly generated by taking the target database and reversing each target sequence. Alternative methods are to use randomized sequences where amino acid sequences are generated using a pre-specified probability distribution [16].

In the concatenated Target-Decoy strategy each spectrum is searched in a single database that is composed of both target and decoy sequences. This involves competition between the best correct peptide sequence, the best incorrect forward peptide sequence, and the best (incorrect) decoy peptide sequence. Hits where the best incorrect decoy peptide sequence is found to be the match are used to estimate the FDR.

In the separate Target-Decoy strategy each spectrum is searched once in the forward database and searched again independently in the decoy database. The distribution of scores from the peptides identified via the decoy database is used to estimate the form of the distribution of incorrectly identified spectra. This approach ignores competition between forward and decoy sequences.

**Figure 6:**
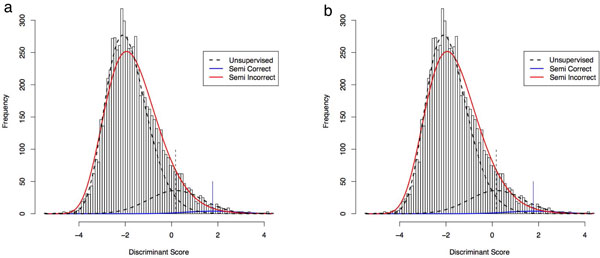
**Semisupervised estimation of parameters**. Semisupervised estimation of parameters of the same distribution of scores as in Figures 2b and 2a. For Charge 3 the slight rightward shift of the Gamma distribution (distribution of scores for incorrect identifications) also encouraged a large rightward shift of the Normal Distribution (distribution of scores for correct identifications). The two vertical lines indicate the means of the Normal distributions. The addition of decoys for Charge 2 allowed to algorithm to learn that most of the identified spectra with scores from 0 to 1 are likely to be incorrect. Without decoys this may have been overlooked.

The semisupervised version of PeptideProphet utilizes the concatenated Target-Decoy strategy by simply combining the target and decoy sequences into the *same *database. The decoy scores are forced to only contribute to the estimation of *α*, *β*, and *γ *of the Gamma distribution. PeptideProphet accomplishes this by assuming any decoy match has a posterior error probability of 1. In the EM-algorithm as described earlier, *p_i _*for any decoy is assumed to be 1 at every iteration. The semisupervised version of PeptideProphet helps estimate the parameters of the Gamma distribution better and thus indirectly improves the estimation of *π*_0_, *μ*, and *σ *as well. As seen in Figure 6a for the case of the Human Plasma dataset the improved estimation of the distributions also increased the separation between the distributions. As seen in Figure 6b the use of decoys helped prevent the possible mistake of having high confidence in scores around the 0 to 1 range.

### PeptideProphet can use a decoy database for semiparametric estimation of the probability distribution

The quality of fit of the Gamma and Normal distributions may rely on how the database is searched (constrained versus unconstrained search) or the search algorithm that is used [12]. As is the case in many statistical modelings, there is no guarantee that the scores of the identified spectra necessarily follow the Gamma and Normal distributions. Previously, decoys were used to estimate parameters of pre-specified distributions. Now we will use decoys for data-dependent estimation of the distributions themselves.

One approach is to estimate the distributional forms using a kernel density (semi-parametric) approach [12] as opposed to maximum likelihood estimation. Kernel density estimates first discretizes the horizontal axis into bins. For a specified bandwidth *h*, the distribution of scores for incorrect identifications is estimated using $p\left(S|h\right)=\frac{1}{n_{0}h}\sum_{i=1}^{n_{0}}K\left(\frac{S-S_{i}}{h}\right)$ where *n*_0 _is the number of decoys, *K *is the Normal density function, and *S_i _*is the score of decoy *i*. The greater the *h *the smoother the function while the smaller the *h *the more rough the function. The parameter *h *can be specified using any method such as using the mean integrated square error. Cross-validation can be used as well. The distribution of scores for correct identifications is estimated in the same fashion as well but using only forward scores. Pseudocode of the semiparametric approach can be seen in Figure 8.

**Figure 7:**
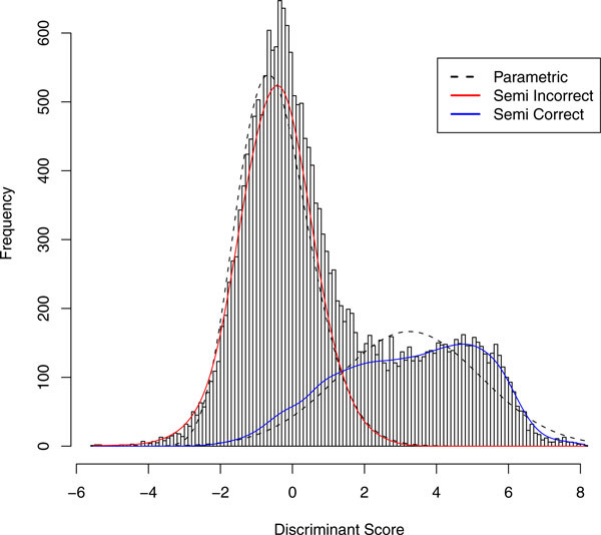
**The Controlled Mixture dataset fit with the basic PeptideProphet and the semiparametric version**. The Controlled Mixture dataset fit with the basic PeptideProphet and the semiparametric version of PeptideProphet utilizing the kernel density estimator. The smoothed estimator allowed for a more fine-tuned fit to the estimated (asymmetric) distribution of the correctly identified spectra.

**Figure 8:**
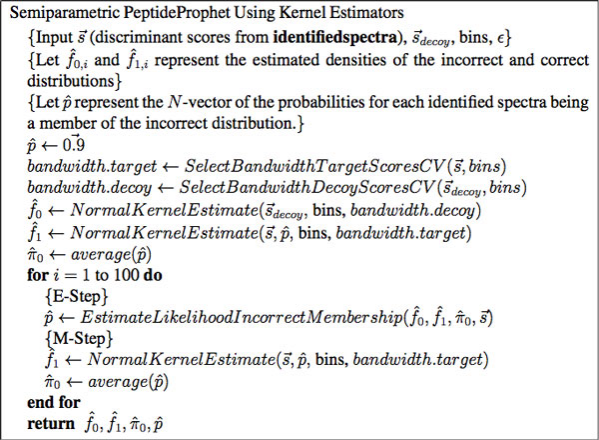
**Pseudocode of the semiparametric version of PeptideProphet**.

An example of this approach can be seen in Figure 7. The parametric fit of the distribution of scores for correct identifications clearly deviates from the Normal curve as the mode of the correct hits is shifted to the right. The semiparametric approach produces a curve that more robustly fits the left-skewed distribution of scores for correct identifications.

To avoid overfitting, this approach should only be used in the cases of strong deviations between the fitted distributions and the observed scores, such as the parametric fit (dashed-lines) in Figure 7. Overfitting typically occurs in experiments with a small number of spectra, such as in Figure 2a. Overfitting can be checked via bootstrapping by seeing if bootstrapped samples do not reflect the same need for a semiparametric fit at certain score values. This can be done via quantile to quantile plots or by checking mean squared errors. If users anticipate good separation, parametric PeptideProphet is often sufficient for practical purposes.

### PeptideProphet can be extended to dynamically estimate the coefficients of the discriminant function from the data

Overlap in the distributions of scores of correct and incorrect identifications can be due to a suboptimal scoring function, which does not discriminate well between the properties of correct and incorrect identifications. This often occurs in cases of constrained searches where the database that is searched is much smaller than the unconstrained search space that was used to find the coefficients in the fixed discriminant function. For additional information on constrained versus unconstrained searches, see [5]. A solution to this is to adapt the discriminant function to each experiment or search approach which can improve the separation between the distribution of scores for incorrect and correct identifications [13].

Pseudocode of the adaptive version of PeptideProphet can be seen in Figure 10. The main step in the algorithm is to update *β*'s from Equations 1 or 2 by extracting identified spectra with high posterior error probabilities and identified spectra with low posterior error probabilities. When retraining the *β*'s the algorithm will randomly sample identified spectra with low posterior error probabilities *I *times and produce *I *different estimates. The average of these *I β*'s is the updated *β*. This entire step is repeated by re-estimating posterior error probabilities and updating *β *until the *β *do not change by a small *ε*.

**Figure 9:**
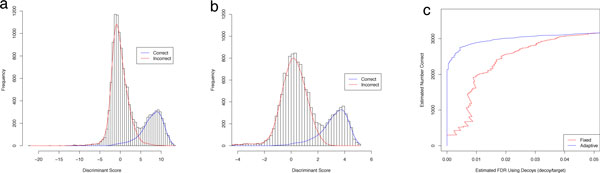
**Semiparametric fits with dynamically estimated coefficients**. Semiparametric fits of the distributions of scores for correct and incorrect identifications on the Controlled Mixture Dataset from a constrained search (tryptic peptides, narrow mass tolerance) using fixed discriminant coefficients (left) versus adaptive discriminant coefficients (center). The right tail of the distribution of scores for incorrect identifications can be seen penetrating the distribution of scores for correct identifications more deeply in the fixed case implying greater discriminative ability when using the adaptive discriminant function. The improved performance of adaptive coefficients can be seen in the plot of the estimated FDR versus the estimated number of significant correctly identified spectra (right). Recall that in this dataset, target scores are assumed correct. The estimated FDR here was estimated by the ratio of the number of decoys to target scores.

**Figure 10:**
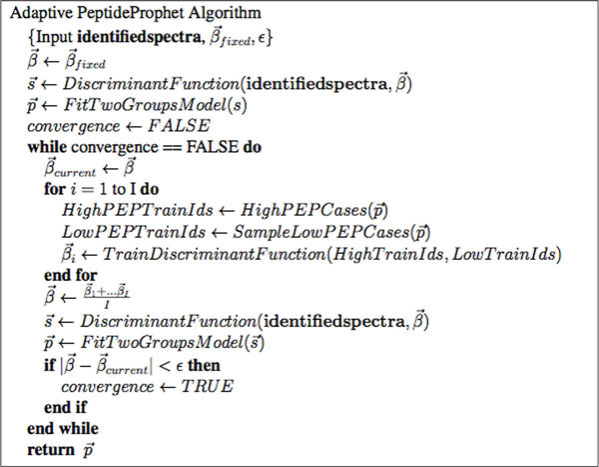
**Pseudocode of the adaptive version of PeptideProphet**.

The improvement of the adaptive discriminant function over the fixed discriminant function for the Controlled Mixture dataset in a constrained search space is displayed in Figure 9. Only tryptic peptides with a narrow mass tolerance were searched.

This approach is also useful for incorporating lower ranked peptide matches (i.e. for a given spectrum, instead of only considering the *best *peptide sequence match, use the new discriminant function to also rescore peptide sequence matches that ranked close to the *best *peptide sequence match). Every time a new discriminant function is estimated (when the *I *${\hat{\beta }}^{\prime }\text{s}$ are averaged) a new summarized score is calculated for the top 5 (can be changed of course) Peptide Matches for every spectrum. The highest scoring peptide-spectrum-match is used in the training of the next discriminant function.

### Implementation of the PeptideProphet in the Trans-Proteomic Pipeline

The Trans-Proteomic Pipieline (TPP) is an open source program developed at the Institute for Systems Biology designed for complete proteomic analysis starting from spectrum identification to protein identification and quantification and can be downloaded from http://sourceforge.net/projects/sashimi/[18]. In this section we assume that search results have already been converted to pepXML files, which is the standard input for PeptideProphet. A discussion of this can be found at (http://tools.proteomecenter.org/wiki/index.php?title=TPP_Tutorial).

We present an example using the Human Plasma dataset where the spectra are searched through Tandem with the k-score plugin with TPP version 4.4. PeptideProphet automatically models all precursor ion charges and outputs the probability of correct identification. A mixture model using the Normal for the distribution of correct scores and a Gumbel distribution for the distribution of incorrect scores.

**Figure 11:**
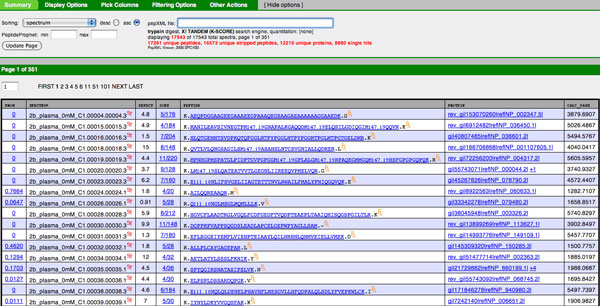
**pepXML viewer from TPP**. The output of PeptideProphet is stored in pepXML format. The pepXML viewer visualizes the content of pepXML and posterior probabilities associated with each identified spectrum.

In Figure 11 of the 17543 identified spectra are listed. The first column lists the probability of correct identification (1 - *PEP*), so numbers close to 1 here are desirable. The remaining columns list, in order, the spectrum label, Tandem expect score, the fraction of ions matched, the peptide sequence match, the protein match, and the calculated neutral peptide mass. In this example any protein label with a "rev" is a decoy. Each hyperlink will lead to additional information. For example, clicking on a peptide sequence will lead to a BLAST search or clicking on the fraction of ions matched will display the observed spectrum.

**Figure 12:**
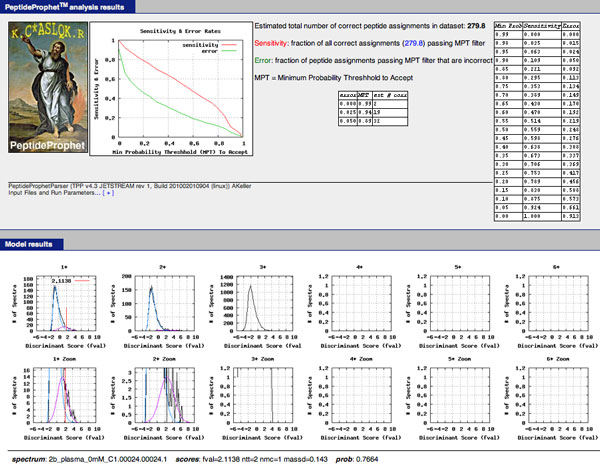
**Scoring results for identified spectra from a PeptideProphet fit in TPP**. PeptideProphet output of sensitivity error analysis and figures of estimated mixture models. The bottom portion shows the fitted curves for different charges. The light blue curves represent the distribution of scores for incorrect identification, purple for correct identifications, and black the sum of the two distributions. The red vertical line also indicates the score for the identified spectra that we clicked on with its additional information at the bottom of the figure.

**Figure 13:**
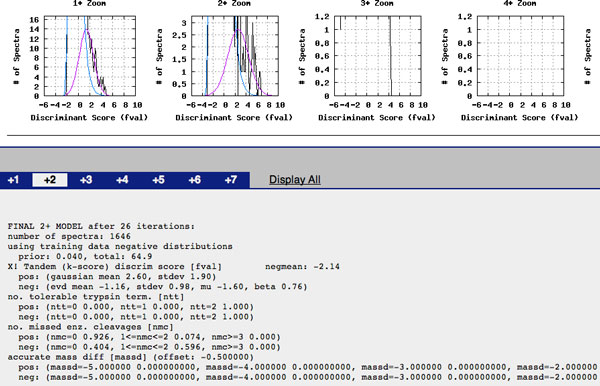
**Parameter estimates for a PeptideProphet fit in TPP**. Estimated parameter values of the PeptideProphet mixture model for charge 2. The parameters of accurate mass difference (Δ*M*) are not fully displayed.

Clicking on 0.7664, or the ninth entry "2b_plasma_0mM_C1.00024.00024.1" on the identified spectra list, results in information of the model fit by PeptideProphet in Figure 12 and the estimated parameter values for charge 2 in Figure 13.

We will now discuss how to use the information in Figures 12 and 13 to estimate the confidence measures discussed previously:

1. False Discovery Rate: estimates of the False Discovery Rate can be obtained three ways. In the upper-right hand corner of Figure 12 estimated False Discovery Rates under the "Error" column is given for 1 - *PEP *values under the "Min Prob" column. In other words, "Min Prob" represents the minimum posterior probability of being correct in order to conclude that an identified spectrum is correct. For example, a "Min Prob" of 0.95 implies that only identified spectra with PEP's lesser than 0.05 are considered correct or that (1 - *PEP*) must be greater than 0.95 to be considered correct.

A second approach is to use the estimated model parameters in Figure 13 to estimate the False Discovery Rate for identified spectra of charge 2. The estimate for (1 - π_0_) is 0.04 which yields an estimate of π_0 _as 0.96. The Normal's (Gaussian) estimated mean *μ *is 2.6 with an estimated standard deviation *σ *of 1.90. The Gumbel's estimated *μ_G _*parameter is -1.16 with an estimated *β *parameter as 0.76. Alternatively, the expected value (mean) of the Gumbel is -1.16 with a standard deviation of 0.98. If the experimenter is not interested in *NTT*, *NMC*, and Δ*M*, for a cutoff score *t*, the estimated FDR can then be estimated by $F\hat{DR}\left(t\right)=\frac{{\hat{p}}_{\text{0}}P\left(S>t|T=0\right)}{{\hat{p}}_{\text{0}}P\left(S>t|T=0\right)+\left(1-{\hat{p}}_{\text{0}}\right)P\left(S>t|T=1\right)}$ where *P*(*S *>*t*|*T *= 0) is found using the Normal distribution and *P*(*S *>*t*|*T *= 1) is found using the Gumbel distribution. Suppose the experimenter wanted to restrict the FDR calculation to identified spectra with only 0 missed cleavages. According to the output in the distribution of correct scores, a randomly selected correctly identified spectra has a 0.926 probability of having 0 missed cleavages and a 0.074 probability of having 1 to 2 missed cleavages. For the distribution of incorrect scores, probabilities are 0.404 and 0.596 for 0 and 1 missed cleavages respectively. The estimated FDR would then be

$$\begin{array}{llll} F\hat{DR}\left(t\right) & =\frac{{\hat{\pi }}_{\text{0}}P\left(S>t|T=0\right)f_{T=0,N\;M\;C}\left(0\right)}{{\hat{\pi }}_{\text{0}}P\left(S>t|T=0\right)f_{T=0,N\;M\;C}\left(0\right)+\left(1-{\hat{\pi }}_{\text{0}}\right)P\left(S>t|T=1\right)f_{T=1,N\;M\;C}\left(0\right)}\; & & \;\\ & =\frac{{\hat{\pi }}_{\text{0}}P\left(S>t|T=0\right)\left(0.404\right)}{{\hat{\pi }}_{\text{0}}P\left(S>t|T=0\right)\left(0.404\right)+\left(1-{\hat{\pi }}_{\text{0}}\right)P\left(S>t|T=1\right)\left(0.926\right)}\; & & \;\\ & \; & \end{array}$$

The calculation takes into account that among the correctly identified spectra, it is estimated that a majority of the identified spectra have 0 missed cleavages.

A third approach of estimating the False Discovery Rate is to download all posterior probabilities, convert them to posterior error probabilities (local false discovery rates) by taking the complement, define a cutoff point *t *and then to calculate $F\hat{D}R\left(t\right)=\frac{\sum_{s_{i}\geq t}PEP_{i}}{\left\{\#s_{i}:s_{i}\geq t\right\}}$.

2. False Positive Rate or p-value: using the Gumbel's estimated parameters, the false positive rate can be found by looking at the tail area.

3. q-value: the q-value at a specific point *δ *can be calculated by estimating the False Discovery Rate at the score value of every identified spectra and then by finding the minimum False Discovery Rate among all scores $s_{i}\leq \delta$.

4. Posterior Error Probability and Local False Discovery Rate: these are most easily found by finding the complement of the values in the first column of Figure 11 or by looking at the complement of "prob" at the bottom center of Figure 12. Note that these probabilities automatically incorporate *NTT*, *NMC*, and Δ*M*. If the experimenter was interested in the posterior error probability of a score independent of *NTT*, *NMC*, and Δ*M*, this can still be calculated using the estimated model parameters.

All inference for semisupervised and semiparametric PeptideProphet cases are identical. Inference would be identical for the adaptive version of PeptideProphet but it is not implemented in TPP at this time but is available from the authors upon request.

Following the execution of PeptideProphet the next step in analysis is often the identification of proteins present in the sample. In this different analysis, the experimental unit changes from being a spectrum to a peptide. TPP can be used to run ProteinProphet, a computational algorithm that can utilize PeptideProphet's estimated probabilities to determine the probability for the presence of proteins in two steps [19]. In the first step the posterior probability of a peptide being correctly identified from PeptideProphet is decreased for peptides that are the only peptide linked to a protein and increased for peptides that are linked to proteins explained by many peptides. In the second step the probability of a protein being in the sample is calculated as the probability that at least one of its associated peptides were identified in the sample. This is $1-\prod_{i}\left(1-p_{i}^{\prime }\right)$ if $p_{i}^{\prime }$ is the adjusted probability of a peptide being in the sample where *i *is indexed from 1 to the number of peptides linked to the protein in question.

## Discussion

PeptideProphet is available for use on the Trans-Proteomic Pipeline with many other database search tools (X!Tandem, MASCOT, OMSSA, Phenyx, ProbID, InsPecT, MyriMatch). The statistical approach of PeptideProphet is generalizable to any database search algorithm that returns a quantitative score for each identified spectrum.

Although we used the Gamma and Normal distributions to model the components of the PeptideProphet model, there are no limitation to the choice of parametric distribution for describing the distributions of scores for incorrect and correct identifications in PeptideProphet. The Gumbel distribution, with parameters *μ *and *β *is another common distribution used for the distribution of scores of incorrect identifications. A generalization of the Gumbel distribution is the Extreme Value Distribution. Additional information, such as the NTT, may be incorporated into the summarized score by using a different machine learning approach instead of a discriminant function. Quantities like the NTT were left out of the summarized discriminant score due to its discrete nature. For example, the logistic regression function would allow discrete and continuous covariates to be transformed into a single summarized score while separating identified spectra with *T *= 0 from identified spectra with *T *= 1.

The Target-Decoy approach used in this manuscript is an approach that pioneered the use of decoys for the estimation of the False Discovery Rate and its results are often compared to other techniques [16]. For the estimation of the False Discovery Rate PeptideProphet and Target-Decoy methods in our experience produce similar results especially when the semisupervised version of PeptideProphet is used as its search approach is similar to the concatenated version of Target-Decoy. In fact, PeptideProphet can be considered as an extension of the concatenated version of Target-Prophet because of its additional modeling objectives. PeptideProphet simply has distributional assumptions and can be used to estimate confidence of individual spectrum identifications or sets of spectrum identifications (local and global FDR estimates) whereas target-decoy is limited to sets (global FDR estimate only). Also, if there is heavy overlap Target-Decoy will outperform basic PeptideProphet but Semisupervised PeptideProphet and Target-Decoy should be similar.

An alternative approach which relaxes the parametric assumptions is the variable component approach which uses an unknown mixture of Gaussians to represent the incorrect and correct distributions of scores [12]. The correct distribution is represented by a mixture distribution of *k*_0 _normal distributions (that may have different means and variances) and the incorrect distribution is represented by a separate mixture distribution of *k*_1 _normal distributions. Parameters *k*_0 _and *k*_1 _are unknown. Each score *s_i _*is a member of either the overall correct or incorrect distributions, but are then further assigned as a member to one of the sub-components of the mixture representing the correct or incorrect distribution. Gibbs sampling is used to estimate the forms of the sub-components (which also suggests the complexity of this approach). Although the variable component and kernel methods perform similarly there are minor computational and modeling issues to consider [12]. The advantages to the variable component method are that: (1) The model is still parametric, which may help reduce the chance of overfitting, (2) Kernel estimation may over fit, especially if the bandwidth is too low, and (3) It does not completely rely on decoys for the negative whereas kernel density estimation uses decoys *only *for estimating the negative distribution. The advantages of the kernel approach are that: (1) The variable component method is much more computationally intensive and more complicated (and thus the Kernel Estimation is less intensive), (2) The variable component method requires the specification of priors, and (3) Kernel estimation is very well known and commonly used.

## Competing interests

The authors declare that they have no competing interests.

## Authors' contributions

K.M. implemented the statistical analysis framework, analyzed the datasets and wrote the manuscript. O.V. supervised the statistical aspects of the work, and wrote the manuscript. A.N. supervised the statistical and the mass spectrometry-based aspects of the work.

## References

[B1] Eng J McCormack A Yates J An approach to correlate tandem mass spectral data of peptides with amino acid sequences in a protein database American Society for Mass Spectrometry 1994 5 976 989 10.1016/1044-0305(94)80016-2 24226387

[B2] Craig R Beavis R TANDEM: matching proteins with tandem mass spectra Bioinformatics 2004 20 9 1466 1467 10.1093/bioinformatics/bth092 14976030

[B3] MacLean B Eng J Beavis R McIntosh M General framework for developing and evaluating database scoring algorithms using the TANDEM search engine Bioinformatics 2006 22 22 2830 2832 10.1093/bioinformatics/btl379 16877754

[B4] Keller A Nesvizhskii A Kolker E Aebersold R Empirical statistical model to estimate the accuracy of peptide identifications made by MS/MS and database search Analytical Chemistry 2002 74 5383 5392 10.1021/ac025747h 12403597

[B5] Nesvizhskii A A survey of computational methods and error rate estimation procedures for peptide and protein identification in shotgun proteomics Journal of Proteomics 2010 73 2092 2123 10.1016/j.jprot.2010.08.009 20816881 PMC2956504

[B6] Whiteaker J Zhang H Eng J Fang R Piening B Feng L Lorentzen T Schoenherr R Keane J Holzman T Fitzgibbon M Lin C Zhang H Cooke K Liu T II DC Anderson L Watts J Smith R McIntosh M Paulovich A Head-to-head comparison of serum fractionation techniques Journal of Proteome Research 2007 6 828 836 10.1021/pr0604920 17269739

[B7] Choi H Nesvizhskii A Semisupervised model-based validation of peptide identifications in mass spectrometry-based proteomics Journal of Proteome Research 2008 7 254 265 10.1021/pr070542g 18159924

[B8] Klimek J Eddes J Hohmann L Jackson J Peterson A Letarte S Gafken P Katz J Mallick P Lee H Schmidt A Ossola R Eng J Aebersold R Martin D The standard protein mix database: a diverse data set to assist in the production of improved peptide and protein identification software tools Journal of proteome research 2007 7 96 103 17711323 10.1021/pr070244j PMC2577160

[B9] Storey J A direct approach to false discovery rates Journal of the Royal Statistical Society. Series B 2002 64 3 479 498 10.1111/1467-9868.00346

[B10] Efron B Microarrays, empirical Bayes and the two-groups model Statistical Science 2008 23 1 22 10.1214/07-STS236

[B11] Kall L Storey J MacCoss M Posterior error probabilities and false discovery rates: two sides of the same coin Journal of Proteome Research 2008 7 40 44 10.1021/pr700739d 18052118

[B12] Choi H Ghosh D Nesvizhskii A Statistical validation of peptide identifications in large-scale proteomics using the target-decoy database search strategy and flexible mixture modeling Journal of Proteome Research 2008 7 286 292 10.1021/pr7006818 18078310

[B13] Ding Y Choi H Nesvizhskii A Adaptive discriminant function analysis and reranking of MS/MS database search results for improved peptide identification in shotgun proteomics Journal of Proteome Research 2008 7 4878 4889 10.1021/pr800484x 18788775 PMC3744223

[B14] Dempster A Laird N Rubin D Maximum likelihood from incomplete data via the EM algorithm Journal of the Royal Statistical Society. Series B 1977 39 1 38 http://www.jstor.org/discover/10.2307/2984875?uid=3738032&uid=2&uid=4&sid=21101269442551

[B15] Storey J The positive false discovery rate: a Bayesian interpretation and the q-value Annals of Statistics 2003 31 6 2013 2035 10.1214/aos/1074290335

[B16] Elias J Gygi S Target-decoy search strategy for increased confidence in large-scale protein identifications by mass spectrometry Nature Methods 2007 4 3 207 214 10.1038/nmeth1019 17327847

[B17] Käll L Storey J MacCoss M Noble W Assigning significance to peptides identified by tandem mass spectrometry using decoy databases Journal of Proteome Research 2008 7 29 34 10.1021/pr700600n 18067246

[B18] Deutsch E Mendoza L Shteynberg D Farrah T Lam H Tasman N Sun Z Nilsson E Pratt B Prazen B Eng JK Martin DB Nesvizhskii AI Aebersold R A guided tour of the Trans Proteomic Pipeline Proteomics 2010 10 1150 1159 10.1002/pmic.200900375 20101611 PMC3017125

[B19] Nesvizhskii A Keller A Kolker E Aebersold R A statistical model for identifying proteins by tandem mass spectrometry Analytical Chemistry 2003 75 http://pubs.acs.org/doi/abs/10.1021/ac0341261 10.1021/ac0341261 14632076

